# Nucleotide-Binding Oligomerization Domain-Like Receptor 3 Deficiency Attenuated Isoproterenol-Induced Cardiac Fibrosis via Reactive Oxygen Species/High Mobility Group Box 1 Protein Axis

**DOI:** 10.3389/fcell.2020.00713

**Published:** 2020-08-11

**Authors:** Chen Liu, Tongtong Hu, Zhulan Cai, Qingwen Xie, Yuan Yuan, Ning Li, Saiyang Xie, Qi Yao, Jinhua Zhao, Qing Qing Wu, Qizhu Tang

**Affiliations:** ^1^Department of Cardiology, Renmin Hospital of Wuhan University, Wuhan, China; ^2^Hubei Key Laboratory of Metabolic and Chronic Diseases, Wuhan, China

**Keywords:** nucleotide-binding oligomerization domain-like receptor 3, cardiac fibrosis, reactive oxygen species, high mobility group box 1 protein, heart failiure

## Abstract

Nucleotide-binding oligomerization domain-like receptor 3 (NLRP3) is involved in fibrosis of multiple organs, such as kidney, liver, lung, and the like. However, the role of NLRP3 in cardiac fibrosis is still controversial and remains unclear. The study aims to investigate the role of NLRP3 on cardiac fibrosis induced by isoproterenol (ISO). *In vivo*, NLRP3 knockout and wild-type mice were subcutaneously injected with ISO to induce the cardiac fibrosis model. The results showed that NLRP3 deficiency alleviated the cardiac fibrosis and inflammation induced by ISO. *In vitro*, neonatal rat ventricular myocytes (NRVMs) and primary adult mouse cardiac fibroblasts of NLRP3 knockout and wild-type mice were isolated and challenged with ISO. Adenovirus (Ad-) NLRP3 and small interfering RNAs targeting NLRP3 were used to transfect NRVMs to overexpress or knockdown NLRP3. We found that NLRP3 could regulate high-mobility group box 1 protein (HMGB1) secretion via reactive oxygen species production in NRVMs and the HMGB1 secreted by NRVMs promoted the activation and proliferation of cardiac fibroblasts. Thus, we concluded that the NLRP3/reactive oxygen species/HMGB1 pathway could be the underlying mechanism of ISO-induced cardiac fibrosis.

## Introduction

Cardiac fibrosis refers to the imbalance of extracellular matrix (ECM) production and degradation and excessive deposition and abnormal distribution of collagen, which is closely related to various cardiovascular diseases, such as hypertension, chronic heart failure (HF), hypertrophic cardiomyopathy, dilated cardiomyopathy, viral myocarditis, etc. and is a potential risk factor for sudden cardiac death. Currently, cardiac fibrosis has been found to relate with renin–angiotensin–aldosterone system, growth factors, oxidative stress, inflammatory factor, endothelial function obstacles, intracellular calcium ion, and so on (Gyongyosi et al., 2017). However, the specific pathogenesis of cardiac fibrosis remains unclear, and the therapies for cardiac fibrosis are not as effective as expected.

Inflammation is one of the major pathophysiological mechanisms in the process of cardiac fibrosis. There is a dynamic interaction between inflammation and fibrosis in various precursors of HF. Virtually any cardiac injury, such as ischemia or infection, can initiate an inflammatory response and trigger several inflammatory pathways in the heart (Suthahar et al., 2017). Pattern recognition receptors, which included Toll-like receptors (TLRs), retinoic acid-inducible gene I-like receptors, nucleotide-binding oligomerization domain (NOD)-like receptors, and C-type lectin receptors, are widely expressed in myocardial tissues. They can initiate host inflammation and are essential to recognize infectious or dangerous foreign patterns, collectively termed as pathogen-associated molecular patterns (PAMPs) and danger-associated molecular patterns (DAMPs). Targeting the cardiac DAMPs and DAMPs, such as TLR2–9, advanced glycation end products receptors (RAGE), NOD1, NOD2, etc., has been reported to exert potentially controlling inflammatory and anti-fibrosis effect in the myocardial response to injury (Turner, 2016).

NOD-like receptor 3 (NLRP3) belongs to the NOD-like receptor family and is a tripartite protein that contains an amino-terminal pyrin (PYD domain), a central NACHT domain and a carboxy-terminal leucine-rich repeat domain. NLRP3 inflammasome consists of a sensor (NLRP3), an adaptor (an apoptosis-associated speck-like protein containing a CARD, ASC), and an effector (caspase 1), which is the one of the most extensively studied inflammasome that can induce a sterile inflammatory response in various diseases. The carboxy-terminal leucine-rich repeat domain recognizes the specific ligand, resulting in the assembly of the NLRP3/ASC/caspase-1 precursor protein complex; then, the caspase-1 precursor self-cleaves into an activated form, which in turn cleaves pro-interleukin (IL)-1β and pro-IL-18 (Swanson et al., 2019). In addition to leukocytes, NLRP3 is also expressed in a variety of non-hematopoietic cell types within solid organ systems, including the heart, but its role in non-professional immune cells remains unclear (Mezzaroma et al., 2011; Sandanger et al., 2013). Studies have found that NLRP3 played a significant role in atherosclerosis, acute myocardial infarction, ischemia–reperfusion, diabetes, and pressure overload-induced cardiac remodeling (Mezzaroma et al., 2011; Luo et al., 2014; Li F. et al., 2018). Fibrosis is an important component of cardiac remodeling, and the role of NLRP3 in cardiac fibrosis is not fully understood. In this study, we used the NLRP3-knockout (KO) mice to investigate the mechanism of NLRP3 in the isoproterenol (ISO)-induced cardiac fibrosis model and aimed to provide a new approach for the prevention and treatment of cardiac fibrosis and HF in the future.

## Materials and Methods

### Animals and Experimental Design

All experimental animal procedures in our study were performed following the Care and Use of Laboratory Animals published by the National Institutes of Health Guide (National Institutes of Health publications no. 8023, revised 1978) and approved by the Animal Care and Use Committee of Renmin Hospital of Wuhan University (approval number: WHRM−2018W05). Male C57/B6 mice (8–10 weeks) weighing 22.5 ± 2.5 g were purchased from the Beijing Vital River Laboratory Animal Technology Co., Ltd. (Beijing, China). NLRP3−KO (stock no. 021302) mice were obtained from the Jackson Laboratory. Mice were injected subcutaneously with ISO or equal volumes of saline for 14 days (10 mg/kg for 3 days and 5 mg/kg for 11 days) (Jiang et al., 2017; Li N. et al., 2018). The animals were randomly divided into four groups (12 per group): wild-type (WT)-Sham, KO-Sham, WT-ISO, and KO-ISO. Two weeks after ISO or saline treatment, echocardiography was performed on anesthetized mice (continuous inhalation of 1.5–2.0% isoflurane). After echocardiography, the mice were euthanized by cervical dislocation, and the hearts and lungs of the mice were harvested and weighed.

### Echocardiography Measurement

To assess the cardiac function of mice, we used the MyLab 30CV ultrasound (Biosound Esaote) with a 10 MHz linear array ultrasound transducer for echocardiography measurements. The parameters, including left ventricular ejection fraction, left ventricular fractional shortening, left ventricular end−diastolic diameter (LVEDD), left ventricular end−systolic diameter (LVESD), and left ventricular end−diastolic posterior wall dimension were recorded.

### Histological Analysis and Immunohistochemistry

The harvested mouse hearts were fixed in the 10% formalin overnight and dehydrated, then embedded in paraffin, after transverse sectioning into 4–5 μm sections. The hematoxylin and eosin, picrosirius red, and immunohistochemical staining methods were performed as previously described (Wu et al., 2016; Wu Q. Q. et al., 2018). Antibody against NLRP3 (ab214185, Abcam, Cambridge, United Kingdom), CD68 (ab125212, Abcam), or CD45 (ab25603, Abcam) were used as the primary antibodies.

### Neonatal Rat Ventricular Myocyte Culture and Treatment

Neonatal rat ventricular myocytes (NRVMs) were isolated and cultured as our previous study (Wu Q. Q. et al., 2018). NRVMs were seeded in 6- or 24-well plates with 1% bromodeoxyuridine for 48 h. Adenovirus (Ad-) NLRP3s (multiplicity of infection = 50) obtained from Vigene Bioscience (Rockville, MD, United States) were transfected into NRVMs for 6 h to overexpress NLRP3. Ad-negative control (NC) was used as a non-targeting control. Small interfering RNAs targeting NLRP3 (siNLRP3) (Ruibo Bio, Guangzhou, China) was used to knock down NLRP3 according to the manufacturer’s protocol. The sequence of si-R-NLRP3 was GCTTCAGCCACATGACTTT. And then, the cells were treated with ISO (10 μM) or phosphate-buffered saline (PBS) for 24 h. N-acetyl-L-cysteine (NAC, 2 mM) was obtained from Sigma-Aldrich and used to inhibit oxidative stress.

### Primary Adult Mouse Cardiac Fibroblasts Culture and Treatment

CFs were isolated and cultured, as described previously (Li F. et al., 2018). The CFs passaged to the 2–4th generation were used for the experiment. Cells were seeded in 6- or 24-well plates and stimulated with ISO (10 μM) or PBS to determine the effect of NRVMs supernatant on the activation and proliferation of CFs. We cultured CFs in the supernatant of NRVMs treated with ISO and siNLRP3 or Ad-NLRP3 for 24 h. Different concentrations (0, 2, 4, 8, and 16 ng/ml) of recombinant human high-mobility group box 1 protein (HMGB1; Biolegend, San Diego, United States) were used to treat CFs in the cell proliferation experiment. The concentration of 16 ng/ml HMGB1 was used for further study, and glycyrrhizic acid (MCE, New Jersey, United States) was used to antagonize HMGB1.

### ELISA Assay

Mouse HMGB-1 Assay Kit (Novus Biologicals, Colorado, United States) and Rat HMGB1 ELISA Kit (Bioss, Beijin, China) were used to detect the serum and cell supernatant HMGB1 content in each group. Briefly, 100 μl standard working solution of different concentrations and samples was added to each well of a 96-well plate and incubated for 90 min at 37°C. After incubation, the solution was discarded. Biotinylated Detection Ab working solution (100 μl) was added to each well immediately and incubated for 1 h at 37°C, then washed three times. Horseradish peroxidase conjugate working solution (100 μl) was added to each well and incubated for 30 min at 37°C and washed three times. Substrate reagent (90 μl) was added to each well and incubated for 15 min at 37°C. At last, 50 μl stop solution was added to each well, and the optical density (OD) value was determined by a microplate reader set to 450 nm.

### Cell Counting Kit-8

Cell Counting Kit-8 (CCK-8) (Beyotime Institute of Biotechnology, Shanghai, China) was used to determine the proliferation of CFs stimulated by different concentrations of HMGB1. Briefly, 10 μl CCK-8 was added to each well and incubated for 1 h in a 37°C incubator, then detected the absorbance at 450 nm with a microplate reader. The cell survival rate (%) = OD treatment/OD control.

### Reactive Oxygen Species Detection

Reactive oxygen species (ROS) Assay Kit (Beyotime Institute of Biotechnology, Shanghai, China) was used for intracellular ROS detection according to the manufacturer’s instructions. Briefly, after the cell culture medium was removed, 1 ml of dichlorofluorescein diacetate diluted 1:1,000 with a serum−free medium was added to a six−well plate and incubated for 30 min at 37°C in a cell incubator, then washing the cells three times with PBS to remove dichlorofluorescein diacetate that did not enter the cells, and observing the ROS immunofluorescence under a laser confocal microscope.

### Immunofluorescence

Immunofluorescence staining was carried out as our previous research (Wu et al., 2016). In brief, cardiac tissue sections were processed for deparaffinization, rehydration, and antigen retrieval with sodium citrate. Then, primary HMGB1 (ab79823, Abcam) antibody was applied at 4°C overnight after blocking with 10% sheep serum; goat anti−rabbit (LI−COR) was used as the secondary antibody incubated for 60 min at 37°C. The cell coverslips were fixed with 4% formaldehyde and permeabilized in 0.2% Triton X-100, then blocking with 10% sheep serum. Antibody against α-smooth muscle actin (α-SMA; ab5694, Abcam), proliferating cell nuclear antigen (PCNA; sc-7907, Santa Cruz, CA, United States), or HMGB1 (ab79823, Abcam) were incubated at 4°C overnight; the remaining steps were the same as tissue staining.

### Quantitative Real-Time Reverse Transcription PCR and Western Blot

TRIzol (Invitrogen, CA, United States) was used for total RNA extraction of mouse cardiac tissue or cells. Then, the reverse transcription and real-time PCRs were performed as previously reported (Li F. et al., 2018) and normalized to the amount of glyceraldehyde 3-phosphate dehydrogenase gene expression. The tissue and cell protein were extracted by the Nuclear and Cytoplasmic Protein Extraction Kit (Beyotime Institute of Biotechnology) according to the manufacturer’s instructions. Western blots were performed according to our previous work (Wu Q. Q. et al., 2018). Primary antibodies, including NLRP3 (ab214185, Abcam), HMGB1 (ab79823, Abcam), glyceraldehyde 3-phosphate dehydrogenase (2118, CST, MA, United States), Lamin B (ab16048, Abcam), β-actin (ab8227, Abcam), nicotinamide adenine dinucleotide phosphate (NADPH) oxidase (NOX) 2 (ab129068, Abcam), NOX4 (ab154244, Abcam), and SOD2 (ab68155, Abcam) were used in Western blot.

### Data and Statistical Analysis

The data were analyzed in one-way ANOVA, followed by a Tukey’s *post hoc* test among multiple groups. Comparisons between two groups were performed using Student’s unpaired *t*-test. Statistical significance was assigned at *P* < 0.05. All statistical analyses were performed by SPSS 23.0 software.

## Results

### Nucleotide-Binding Oligomerization Domain-Like Receptor 3 Was Upregulated in Isoproterenol-Induced Cardiac Remodeling

**FIGURE 1:**
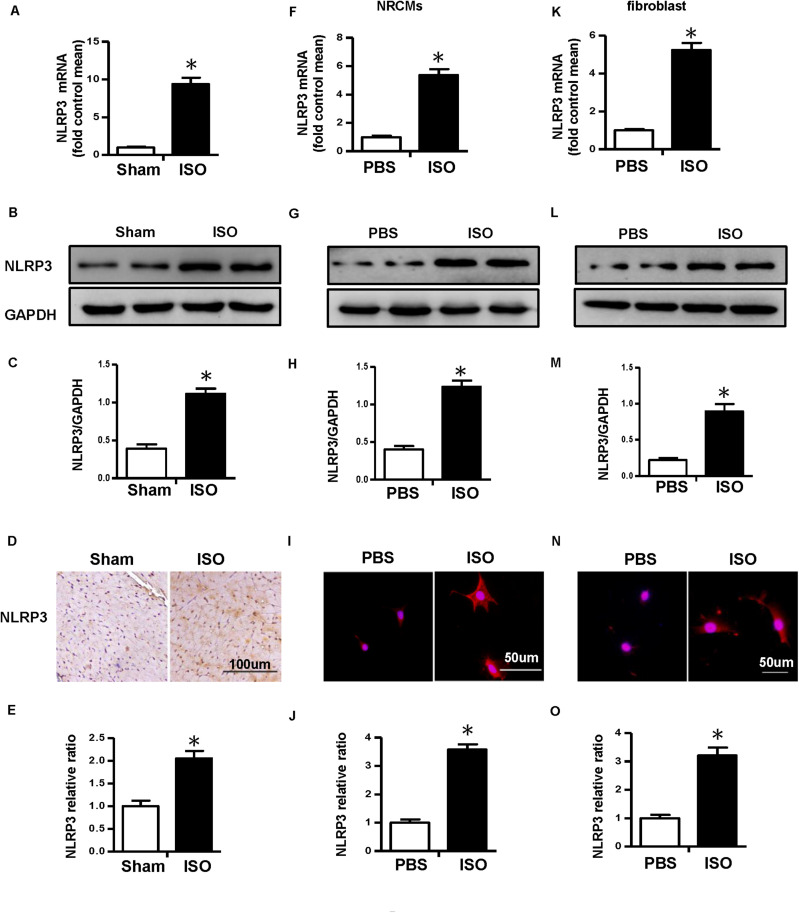
Nucleotide-binding oligomerization domain-like receptor 3 (NLRP3) was upregulated in isoproterenol (ISO)-induced cardiac remodeling. **(A–C)** Messenger RNA (mRNA) and protein expression level and quantitative results of NLRP3 in mouse heart induced by ISO (*n* = 6). **(D–E)** Immunohistochemical staining and quantification of NLRP3 in mouse heart treated with ISO or equal volumes of saline for 14 days (*n* = 6). *P < 0.05 vs. sham group. **(F–H)** mRNA and protein expression level and quantitative results of NLRP3 in neonatal rat ventricular myocytes challenged with ISO (*n* = 6). **(I,J)** Immunofluorescence staining and quantification of NLRP3 in neonatal rat ventricular myocytes stimulated with ISO or phosphate-buffered saline (PBS) for 24 h (*n* = 6). **(K–M)** mRNA and protein expression level and quantitative results NLRP3 in cardiac fibroblasts challenged with ISO (*n* = 6). **(N,O)** Immunofluorescence staining and quantification of NLRP3 in cardiac fibroblasts stimulated with ISO or PBS for 24 h (*n* = 6). *P < 0.05 vs. PBS group.

First, we detected the messenger RNA (mRNA) and protein expression level of NLRP3 in ISO-treated mice heart *in vivo* and ISO-challenged NRVMs and CFs *in vitro*. The result of real-time PCR and Western blot showed that NLRP3 increased in the mice treated with ISO *in vivo* (Figures 1A–C). Additionally, the NLRP3 expression level also increased in the NRVMs (Figures 1F–H) and CFs (Figures 1K–M) challenged with ISO. The immunohistochemistry and immunofluorescence staining results were consistent with the Western blot (Figures 1D,E,I,J,N,O).

### Nucleotide-Binding Oligomerization Domain-Like Receptor 3 Deficiency Alleviated the Cardiac Fibrosis and Inflammation and Improved Cardiac Function Induced by Isoproterenol

**FIGURE 2:**
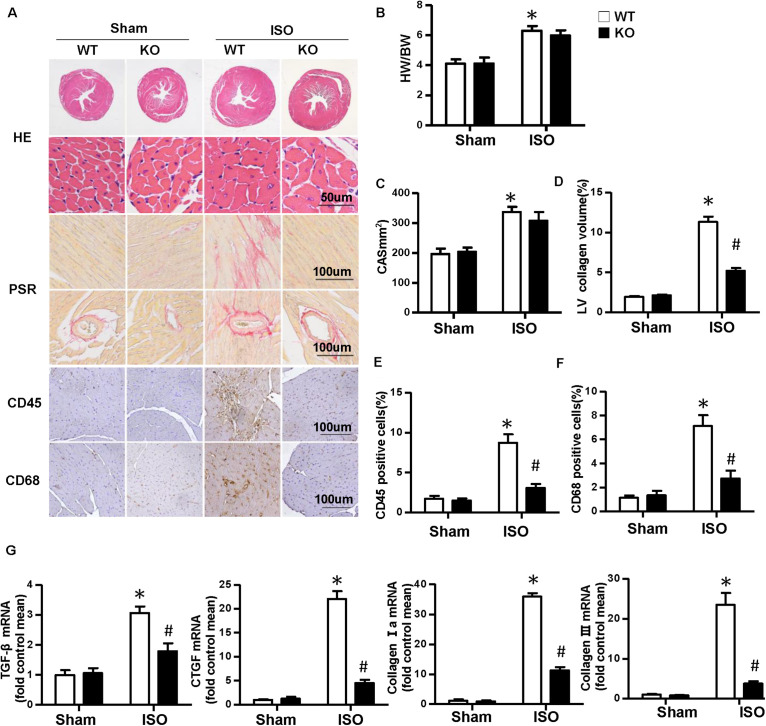
Nucleotide-binding oligomerization domain-like receptor 3 (NLRP3) deficiency alleviated the cardiac fibrosis and inflammation induced by isoproterenol. **(A)** Images of hematoxylin and eosin, picrosirius red, and immunohistochemistry staining for CD45, CD68 of each group (*n* = 6). **(B)** Statistical results for the heart weight/body weight (*n* = 12). **(C)** Cross-sectional area results of cardiomyocyte in the indicated group (n = 6, 100 + cells per group). **(D–F)** Quantitative results of picrosirius red, CD45, CD68 staining (*n* = 6). **(G)** Relative messenger RNA levels of transforming growth factor β, connective tissue growth factor, collagen Ia, and collagen III in mice heart (*n* = 6). *P < 0.05 vs. sham + wild type, ^#^*P* < 0.05 vs. isoproterenol + wild type.

**FIGURE 3:**
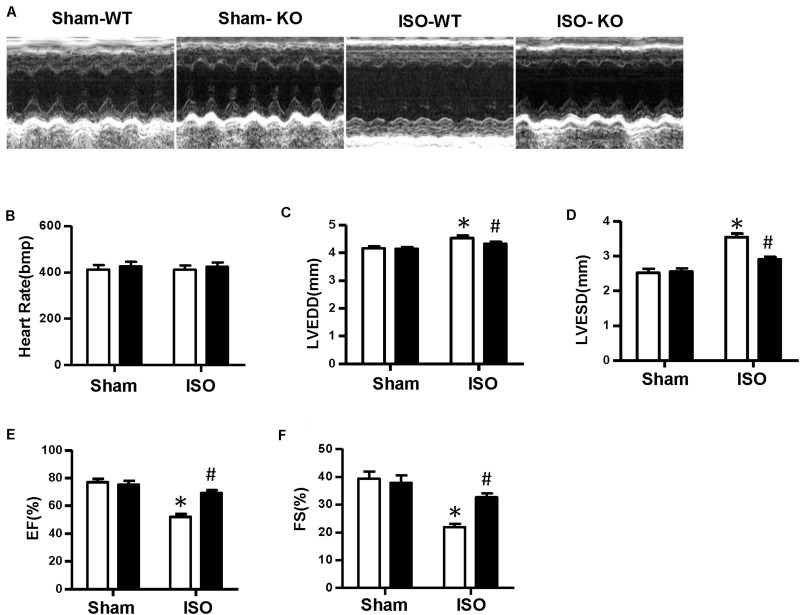
Nucleotide-binding oligomerization domain-like receptor 3 deficiency improved cardiac function induced by isoproterenol. **(A)** Representative echocardiographic images of each group. **(B–F)** Echocardiographic result of heart rate, left ventricular end–diastolic diameter, left ventricular end–systolic diameter, fractional shortening, and ejection fraction in the indicated group (*n* = 8). *P < 0.05 vs. sham + wild type, ^#^*P* < 0.05 vs. isoproterenol + wild type.

Multiple complex pathological mechanisms involved in cardiac remodeling contribute to altering the structure and function of the heart. Among them, fibrosis and inflammation are significant features in the process of cardiac remodeling (Kehat and Molkentin, 2010; Schirone et al., 2017). Here, we investigate the effect of NLRP3 on ISO-induced cardiac remodeling. NLRP3-KO mice were used in the animal experiment. As shown in Figures 2A–C, the heart weight/body weight ratio and cardiomyocyte cross−sectional area increased after ISO treatment, however, NLRP3 deficiency did not affect the heart weight/body weight ratio and cardiomyocyte dimensions. Picrosirius red staining was used to detect the collagen deposition of the heart; we found that interstitial and perivascular fibrosis exacerbated in the WT mice treated with ISO; NLRP3 deficiency ameliorated the fibrosis process in mice challenged with ISO (Figures 2A,D) as well as the transcription levels of fibrosis markers—transforming growth factor β, connective tissue growth factor (CTGF), collagen I, and collagen III (Figure 2G). Additionally, CD45 and CD68 immunohistochemistry staining that demonstrated the infiltrated leukocytes and macrophages were increased after ISO treatment; NLRP3 deficiency decreased infiltration of inflammatory cells (Figures 2A,E,F). Echocardiography was used to assess cardiac function. Consistent with our previous research, ISO-treated mice exhibited worse cardiac function with increased LVEDD and LVESD and decreased ejection fraction and fractional shortening; NLRP3 deficiency prevented the deterioration of cardiac function induced by ISO (Figures 3A–F).

### Nucleotide-Binding Oligomerization Domain-Like Receptor 3 Deficiency Did Not Influence Activation and Proliferation of Cardiac Fibroblasts

**FIGURE 4:**
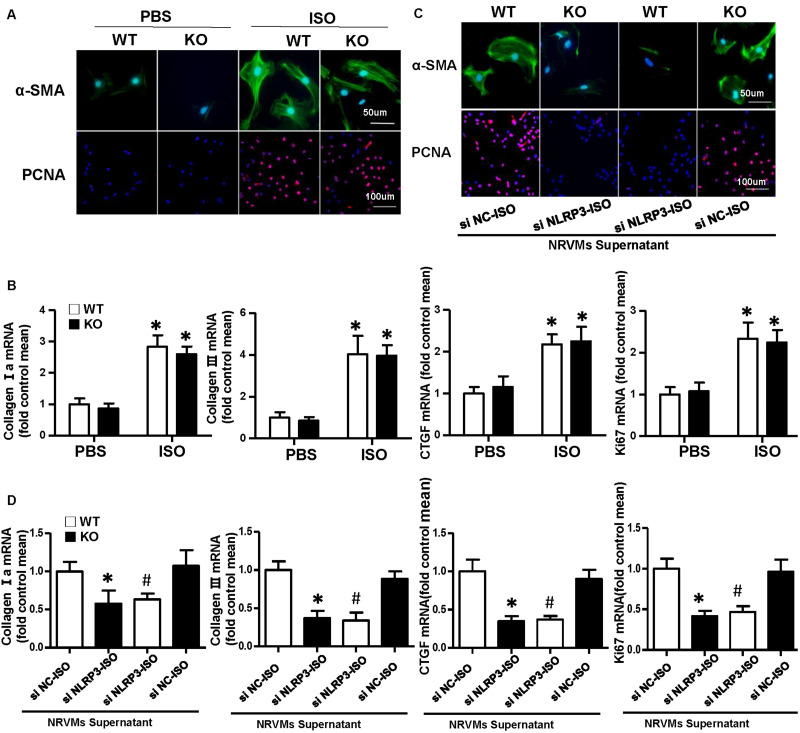
Nucleotide-binding oligomerization domain-like receptor 3 (NLRP3) deficiency did not influence activation and proliferation of cardiac fibroblasts (CFs), but coculture CFs with the supernatant of neonatal rat ventricular myocytes enhanced the activation and proliferation of it *in vitro*. **(A)** Immunohistochemical staining of α-smooth muscle actin and proliferating cell nuclear antigen in CFs of wild-type (WT) and knockout mice stimulated by isoproterenol (ISO) (*n* = 6). **(B)** Real-time PCR analyses of fibrotic markers (collagen I, collagen III, connective tissue growth factor, and Ki67) in each group (*n* = 6). *P < 0.05 vs. WT-phosphate-buffered saline group; ^#^*P* < 0.05 vs. WT-ISO group. **(C)** Immunohistochemical staining of α-smooth muscle actin and proliferating cell nuclear antigen in CFs cocultured with the supernatant of neonatal rat ventricular myocytes treated with ISO and siNLRP3 or siNC (*n* = 6). **(D)** Real-time PCR analyses of fibrotic markers (collagen I, collagen III, and connective tissue growth factor) and Ki67in the indicated group. *P < 0.05 vs. knockout CFs + si NC-ISO supernatant; ^#^*P* < 0.05 vs. WT CFs + si NC-ISO supernatant.

Cardiac fibroblasts (CFs) exert a significant role in the process of cardiac fibrosis in cardiac remodeling. When the heart is injured, activated CFs begin to proliferate and transdifferentiate into myofibroblasts and secrete collagen and other ECM proteins. To explore the effect of NLRP3 on CFs, we isolated the CFs from WT mice and NLRP3-KO mouse hearts and then treated with ISO for 24 h. As shown in Figure 4A, α-SMA and PCNA immunofluorescence staining showed that NLRP3 deficiency did not influence fibroblast activation and proliferation. Additionally, the transcription levels of collagen I, collagen III, CTGF, and ki67 also shown no significant difference between the WT-ISO and KO-ISO group (Figure 4B).

### Activation and Proliferation of Cardiac Fibroblast Enhanced After Coculture With Neonatal Rat Ventricular Myocytes Supernatant

To further investigate the how NLRP3 regulates cardiac fibrosis, we cocultured the CFs with NRVMs supernatant treated with ISO and siNC or siNLRP3; we found that the α-SMA and PCNA expressions of the CFs were both increased after cocultured with NRVM supernatants treated with ISO + siNC and decreased after cocultured with NRVM supernatants treated with ISO + siNLRP3 (Figure 4C). Additionally, the transcription levels of fibrosis markers (collagen I, collagen III, and CTGF) and ki67 have also shown the same changing trend with the immunofluorescence staining (Figure 4D).

### Nucleotide-Binding Oligomerization Domain-Like Receptor 3 Deficiency Inhibited the Nuclear Translocation and Secretion of High-Mobility Group Box 1 Protein of Cardiomyocytes *in vivo* and *in vitro*

**FIGURE 5:**
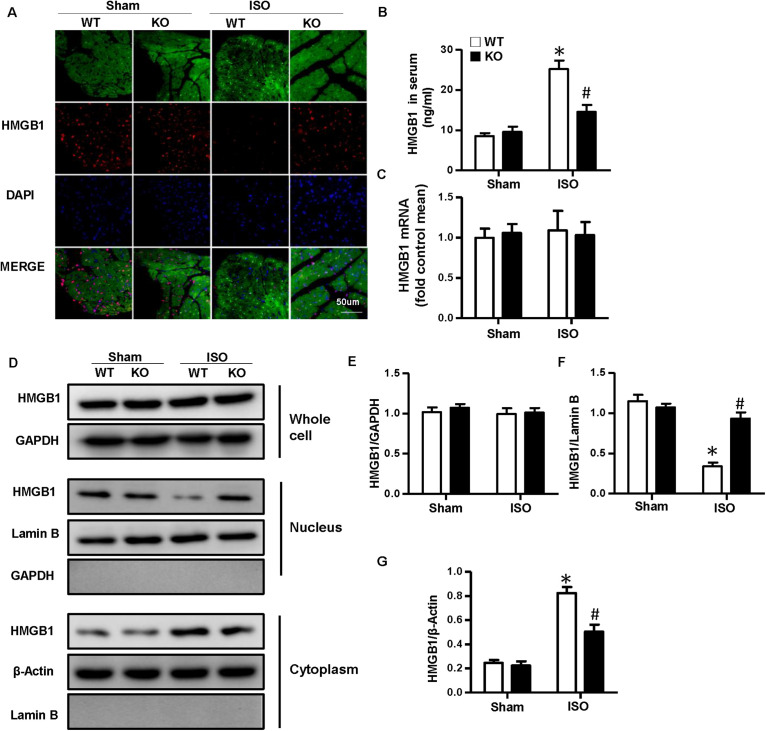
Nucleotide-binding oligomerization domain-like receptor 3 (NLRP3) deficiency inhibited the nuclear translocation and secretion of HMGB1 in neonatal rat ventricular myocytes *in vivo*. **(A)** Immunofluorescence staining of HMGB1 in the mouse heart of each group (*n* = 6). **(B)** Serum HMGB1 content in the indicated group (*n* = 6). **(C)** mRNA expression of HMGB1 in the heart tissue of each group (*n* = 6). **(D–G)** Protein expression of whole-cell, nucleus, and cytoplasm HMGB1 in the heart tissue of each group. Western blot images **(D)** and quantitative results **(E–G)** (*n* = 6). *P < 0.05 vs. sham + WT, ^#^*P* < 0.05 vs. ISO + WT.

**FIGURE 6:**
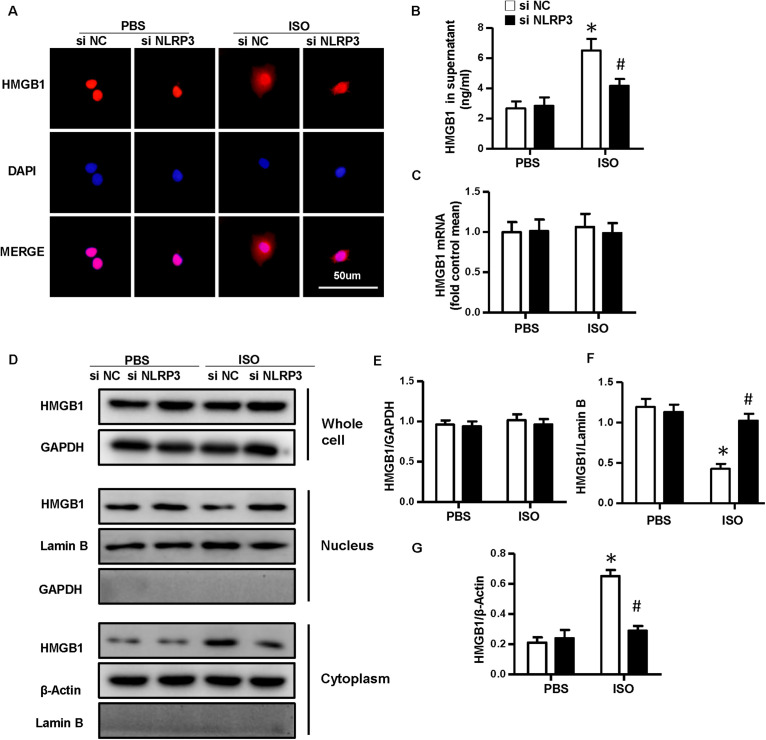
Nucleotide-binding oligomerization domain-like receptor 3 (NLRP3) deficiency inhibited the nuclear translocation and secretion of HMGB1 in neonatal rat ventricular myocytes *in vitro*. **(A)** Immunofluorescence staining of HMGB1 in neonatal rat ventricular myocytes treated with siNLRP3 or siNC and ISO or phosphate-buffered saline (PBS) (*n* = 6). **(B)** HMGB1 content in neonatal rat ventricular myocytes supernatant of each group (*n* = 6). **(C)** Messenger RNA expression of HMGB1 of each group (*n* = 6). **(D–G)** Protein expression of whole-cell, nucleus, and cytoplasm HMGB1 in neonatal rat ventricular myocytes treated with siNLRP3 and ISO or PBS. Western blot images **(D)** and quantitative results **(E–G)**. *P < 0.05 vs. PBS + siNC, ^#^*P* < 0.05 vs. ISO + siNC.

As NLRP3-KO did not affect the activation and proliferation of CFs after ISO treatment *in vitro*, NLRP3 deficiency could indeed alleviate cardiac fibrosis *in vivo*, and the activation and proliferation of CFs could be enhanced after coculture with NRVMs supernatant. Here, we hypothesized that cardiomyocyte–fibroblast interaction might contribute to the cardiac fibrosis induced by ISO. To test our hypothesis, we detected the serum HMGB1 content and found that the HMGB1 level in serum increased in the WT-ISO group and dramatically decreased in the KO-ISO group (Figure 5B). Also, the immunofluorescence staining and Western blot have shown the nuclear translocation of HMGB1 reduced in the NLRP3-KO mice after ISO treatment (Figures 5A,D,F,G). However, the mRNA and total protein expression of HMGB1 was not changed significantly (Figures 5C–E). In the cell experiment part, we isolated the NRVMs and used siNLRP3 to knock down the NLRP3 (Supplementary Figures 1A,B). The results were consistent with the animal experiments. As shown in Figure 6B, HMGB1 level of cell supernatant rises after the 24 h ISO treatment, and NLPR3 knockdown reversed this effect after ISO treatment. Immunofluorescence staining has also shown the nuclear translocation of HMGB1 (Figure 6A). Furthermore, the Western blot has shown that the HMGB1 protein expression level in nuclear decreased in NRVMs treated with ISO and increased in NRVMs treated with ISO + siNLRP3. The HMGB1 protein expression changes in the cytoplasm are opposite to the changes in the nucleus (Figures 6D,F–G), and the mRNA and total protein expression of HMGB1 also was not changed significantly (Figures 6C–E).

### Nucleotide-Binding Oligomerization Domain-Like Receptor 3 Overexpression Enhanced the Nuclear Translocation and Secretion of High-Mobility Group Box 1 Protein of Cardiomyocytes *in vitro*

**FIGURE 7:**
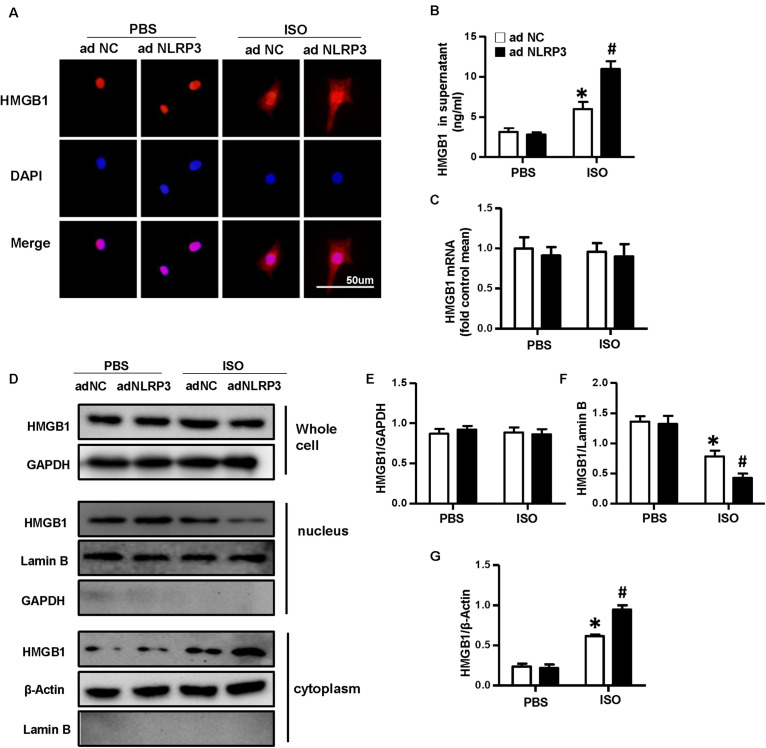
Nucleotide-binding oligomerization domain-like receptor 3 (NLRP3) overexpression enhanced the nuclear translocation and secretion of HMGB1 in neonatal rat ventricular myocytes. **(A)** Immunofluorescence staining of HMGB1 neonatal rat ventricular myocytes treated with Ad-NLRP3 or Ad-NC and ISO or phosphate-buffered saline (*n* = 6). **(B)** HMGB1 content in NRVMs supernatant of each group (*n* = 6). **(C)** Messenger RNA expression of HMGB1 of each group (*n* = 6). **(D–G)** Protein expression of whole-cell, nucleus, and cytoplasm HMGB1 in neonatal rat ventricular myocytes of the indicated group. Western blot images **(D)** and quantitative results **(E–G)** (*n* = 6). *P < 0.05 vs. phosphate-buffered saline + Ad-NC, ^#^*P* < 0.05 vs. ISO + Ad-NC.

To confirm the effect of NLRP3 on HMGB1 nuclear translocation and secretion, we used the Ad-NLRP3 to overexpress the NLRP3 in NRVMs (Supplementary Figures 1C,D). The immunofluorescence staining results indicated the NLRP3 overexpression that enhanced the nuclear translocation of HMGB1 induced by ISO (Figure 7A). The supernatant HMGB1 levels of NRVMs also increased after NLRP3 overexpression when challenged with ISO (Figure 7B). Additionally, the nuclear HMGB1 protein expression levels further decreased, and cytoplasm HMGB1 protein expression level increased in the Ad-NLRP3 + ISO group compared with that in the Ad-NC + ISO group (Figures 7D,F,G). However, the mRNA and total protein expression of HMGB1 was not changed significantly (Figures 7C–E).

### High-Mobility Group Box 1 Protein Secreted by Neonatal Rat Ventricular Myocytes Promoted the Activation and Proliferation of Fibroblasts

**FIGURE 8:**
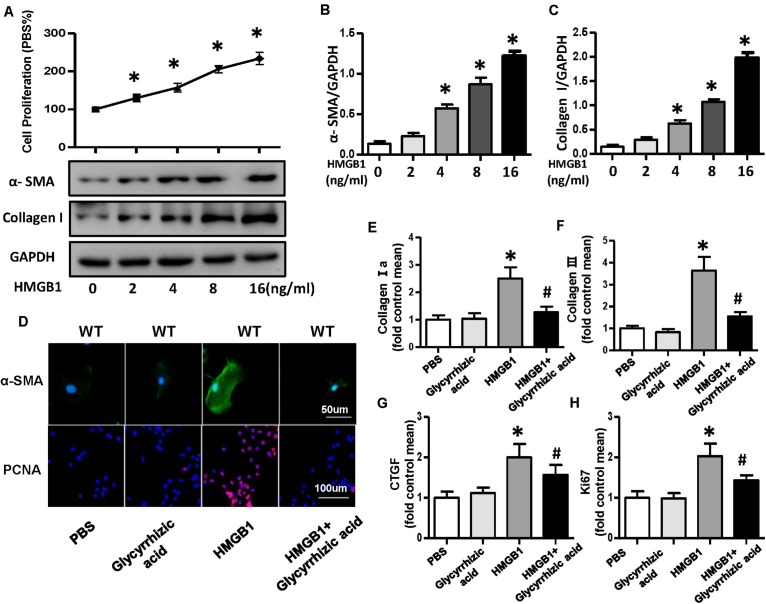
HMGB1 secreted by neonatal rat ventricular myocytes promoted activation and proliferation of cardiac fibroblasts (CFs). **(A–C)** Cell proliferation rate of CFs and protein expression of α-smooth muscle actin and collagen I after the treatment of different concentrations (0, 2, 4, 8, and 16 ng/ml) of HMGB1 (*n* = 5). **(D)** Immunofluorescence staining of α-smooth muscle actin and PCNA of the CFs treated with HMGB1 (16 ng/ml) or HMGB1 inhibitor glycyrrhizic acid (*n* = 6). **(E–H)** Messenger RNA expression levels of collagen I a, collagen III, CTGF, and KI67 in each group (*n* = 6). *P < 0.05 vs. phosphate-buffered saline, ^#^*P* < 0.05 vs. HMGB1.

A growing body of evidence implicated that HMGB1 could act as a DAMP to bind multiple receptors, such as RAGE, TLR2, TLR4, TLR7, and others and could lead to the activation of multiple signaling pathway that end up in regulating cell migration, proliferation, differentiation, and adhesion as well as inflammation and tissue repair (Pellegrini et al., 2019). To further validate whether HMGB1 secreted by NRVMs mediated the proliferation and differentiation of CFs, we used the different concentrations (0, 2, 4, 8, and 16 ng/ml) of recombinant human HMGB1 (carrier-free) to stimulate fibroblast; the result of CCK-8 showed the proliferation rate of CFs increases with the increase of concentration of HMGB1 (Figure 8A). Additionally, the protein expression of α-SMA and collagen I also increased with the concentration of HMGB1 (Figures 8A–C). So, we chose this concentration of 16 ng/ml HMGB1 for the further experiment; we also used HMGB1 inhibitor glycyrrhizic acid; the immunofluorescence staining of α-SMA and PCNA showed obvious activation and proliferation of fibroblast stimulated by HMGB1, which was remarkably reversed after glycyrrhizic acid treatment (Figure 8D). The mRNA expression level of collagen I, collagen III, CTGF, and ki67 also upregulated after HMGB1 stimulation and downregulated after glycyrrhizic acid + HMGB1 treatment (Figures 8E–H).

### Nucleotide-Binding Oligomerization Domain-Like Receptor 3 Affected the High-Mobility Group Box 1 Protein Secretion by Regulating the Production of Reactive Oxygen Species in Neonatal Rat Ventricular Myocytes

**FIGURE 9:**
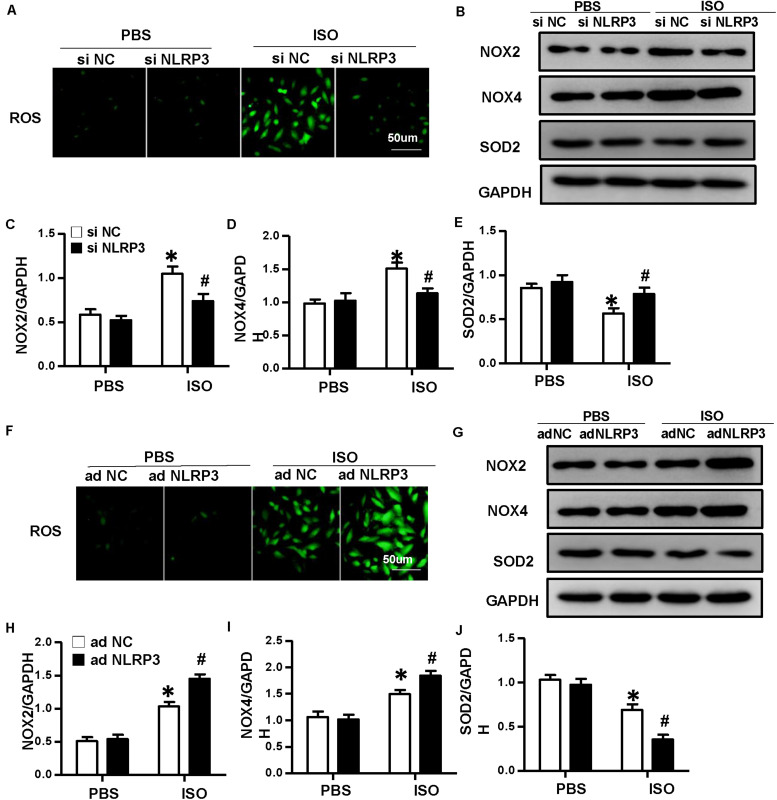
Nucleotide-binding oligomerization domain-like receptor 3 (NLRP3) regulated the production of ROS in neonatal rat ventricular myocytes (NRVMs). **(A)** ROS production of NRVMs transfected with si NLRP3 or si NC and stimulated by ISO or phosphate-buffered saline (PBS) (*n* = 6). **(B–E)** Protein expression of NOX2, NOX4, and SOD2 in NRVMs in the indicated group. Western blot images **(B)** and quantitative results **(C–E)** (*n* = 6). *P < 0.05 vs. PBS + siNC, ^#^*P* < 0.05 vs. ISO + siNC. **(F)** ROS production of NRVMs transfected with Ad-NLRP3 or Ad-NC and stimulated by ISO or PBS (*n* = 6). **(G–J)** Protein expression of NOX2, NOX4, and SOD2 in NRVMs in the indicated group. Western blot images **(G)** and quantitative results **(H–J)** (*n* = 6). *P < 0.05 vs. PBS + Ad-NC, *^#^P* < 0.05 vs. ISO + Ad-NC.

**FIGURE 10:**
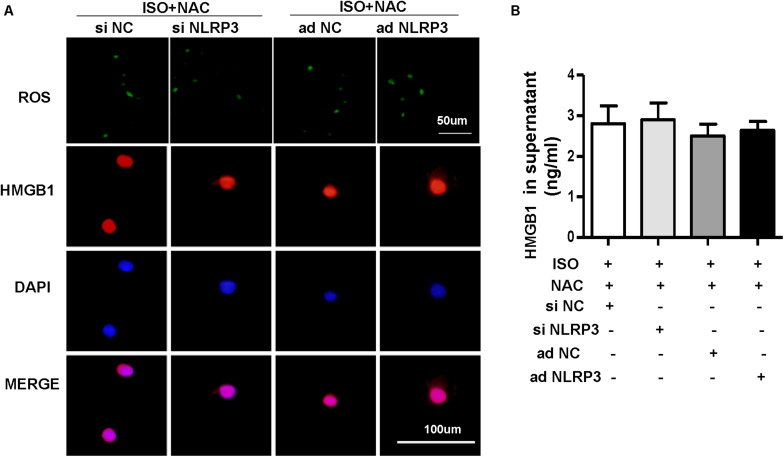
Nucleotide-binding oligomerization domain-like receptor 3 (NLRP3) affected the HMGB1 secretion by regulating the production of ROS in neonatal rat ventricular myocytes (NRVMs). **(A,B)** NRVMs were transfected by small interfering RNAs targeting NLRP3 or Ad-NLRP3 and challenged by ISO or phosphate-buffered saline; N-acetyl-L-cysteine was used to inhibit ROS production. ROS production and immunofluorescence staining of HMGB1 of NRVMs in the indicated group (*n* = 6) **(A)**. HMGB1 content in NRVMs supernatant of each group (*n* = 6) **(B)**.

To explore how NLRP3 mediates HMGB1 secretion, we further detect ROS production and the protein expression of NOX2, NOX4, and SOD2 in NRVMs after knockdown or overexpression of NLRP3 with or without ISO challenge. The result indicated the ROS generation and protein expression of NOX2, NOX4 increase, and SOD2 decrease after treatment with ISO for 24 h; these changes were inhibited by NLRP3 knockdown in NRVMs (Figures 9A–E). Also, we also found that NLRP3 overexpression enhanced ISO-induced upregulation of ROS and protein expression of NOX2 and NOX4 and decreased the SOD2 protein expression in NRVMs (Figures 9F–J). Furthermore, the ROS scavenger NAC was used to determine whether the secretion of HMGB1 was dependent on ROS generation. As a result, NAC suppressed the ISO-induced ROS production and HMGB1 nuclear translation and secretion in NRVMs (Figures 10A,B).

## Discussion

NLRP3 inflammasome is involved in the fibrosis process of various organs or tissue, such as kidney fibrosis (Lorenz et al., 2014), pulmonary fibrosis (Hosseinian et al., 2015), liver fibrosis (Alegre et al., 2017), and cardiac fibrosis (Sandanger et al., 2013; Luo et al., 2014; Li F. et al., 2018). Growing evidence had shown that NLRP3 could trigger the process of fibrosis through NLRP3 inflammasome-dependent or -independent pathway (Artlett and Thacker, 2015). Cardiac fibrosis is a common feature of many pathological cardiac remodeling. Numerous studies have shown that inhibition of the NLRP3 inflammasome pathway exhibited anti-fibrosis effects (Gan et al., 2018; Qiu et al., 2018; Wan et al., 2018; Pan et al., 2019; Gao et al., 2019). However, recently, Li F. et al. (2018) found that NLRP3 deficiency exacerbates cardiac fibrosis and inflammation induced by pressure overload, which was contradictory with the previous studies. To clarify the effect of NLRP3 on cardiac fibrosis, we established an ISO-induced cardiac fibrosis model. ISO can activate the renin–angiotensin system and increase collagen synthesis and is currently the main method used to induce cardiac fibrosis (Ocaranza et al., 2002). Additionally, as a β-adrenergic receptor agonist, ISO can act on both cardiomyocytes and CFs to participate in heart disease occurrence, progression, and prognosis of the disease (Aranguiz-Urroz et al., 2011). In our study, we have demonstrated that NLRP3 deficiency attenuated ISO-induced fibrosis and inflammation *in vivo*. However, CFs themselves that lack NLRP3 did not affect the activation and proliferation of CFs. The underlying mechanism is that NLRP3 could regulate the ROS production that contributed to HMGB1 secretion of cardiomyocytes induced by ISO, and cardiomyocyte-derived HMGB1 could act on CFs and affect their proliferation and activation.

HMGB1 is a widely studied DAMP or alarmin, which belongs to the high-mobility group protein family and expressed in the nucleus of almost all mammalian cells. It can be induced into the cytoplasm and extracellular space and in various cells under many pathophysiological conditions (Goodwin et al., 1973). The location, context, and posttranslational modifications determine the biological activity of HMGB1 (Pellegrini et al., 2019). In the nucleus, HMGB1 acts as a DNA chaperone to regulate DNA repair, transcription regulation, and genome stability (Singh and Dixon, 1990; He et al., 2017). In the cytoplasm, HMGB1 presents at a low level under physiological conditions and mainly functions to regulate autophagic flux (Tang et al., 2010; Huang et al., 2012; Zhu et al., 2015). In the extracellular environment, it possesses a vast variety of functions, including inflammation, migration, invasion, proliferation, differentiation, and tissue regeneration (Pellegrini et al., 2019). The extracellular functions of HMGB1 are mainly mediated by binding with various cellular receptors, which included RAGE (Qian et al., 2019), TLRs (TLR2, 4, 7, and 9) (Coleman et al., 2017; Aucott et al., 2018; Chu et al., 2018), chemokine (C-X-C motif) receptor 4 (Di Maggio et al., 2017) and so on leading to the activation of multiple downstream pathway. In the last years, elevated serum HMGB1 has been found frequently associated with various diseases, especially autoimmune and inflammatory diseases (de Souza et al., 2013; Bobek et al., 2014; Palone et al., 2016; Koutsonikoli et al., 2017), and could represent a possible clinical biomarker in these patients. In heart diseases, the role of HMGB1 is very controversial; studies have suggested that it is a marker of myocardial damage, whereas it also plays a role in tissue repair and regeneration (Pellegrini et al., 2019). It has been reported that NLRP3 inflammasome promoted HMGB1 secretion in vascular smooth muscle cells, and extracellular HMGB1 is a key downstream signal molecule of NLRP3 inflammasome activation (Wang et al., 2018). In our study, we also found that NLRP3 promotes the HMGB1 secretion in ISO-induced NRVMs, and the extracellular HMGB1 could act as a profibrotic factor that mediated the proliferation and differentiation of CFs. However, silencing or overexpressing NLRP3 alone did not influence the nuclear translocation and secretion of HMGB1 in NRVMs. As a kind of pattern recognition receptor, the activation of NLRP3 depends on the perception of “danger signals,” such as DAMP or PAMP in the cells (Swanson and Deng, 2019). Under the stimulation of ISO in NRVMs, a large amount of PAMP was produced in the cell, which enhanced the activation of NLRP3 inflammasome. Overexpressing NLRP3 increased the receptors of those PAMPs, further enhanced NLRP3 inflammasome activation and the nuclear translocation and secretion of HMGB1. However, in the baseline level, maybe lack of danger signaling molecules, Ad-NLRP3 alone did not influence the nuclear translocation and secretion of HMGB1 in NRVMs.

Previous studies have shown that hypoxia-induced ROS could enhance HMGB1 secreted into extracellular in the normal human nasal epithelium cells (Min et al., 2017); ROS could also regulate HMGB1/IL-17A axis to promote the apoptosis in microglial cells (Zhang et al., 2017). Excessive production of ROS itself can cause cell damage; HMGB1 can act as a molecular danger to sense cell damage; therefore, some close connections may exist between them. To further explore how NLRP3 regulates the secretion of HMGB1, we detected the ROS production in NRVMs after overexpression or knockdown of NLRP3. In the present study, we found that NLRP3 overexpression increased the ROS production, and NLRP3 knockdown decreased the ROS production in NRVMs stimulated by ISO, which indicated that NLRP3 could regulate the generation of ROS. However, most of the previous studies have considered that ROS is a redox signaling molecule that could trigger NLRP3 inflammasome activation in the heart (Minutoli et al., 2016; Zhang H. et al., 2018; Zhang X. et al., 2018; Qiu et al., 2019), which were contradictory with our study. In fact, ROS may act as not only a triggering factor to activate NLRP3 inflammasomes but also an effector molecule (Abais et al., 2015); early ROS generation may be a signaling mechanism to activate the formation and activation of NLRP3 due to its action on thioredoxin-interacting protein (TXNIP) binding (Abais et al., 2014) but may not yet have major damage to cell function or activity. After the NLRP3 inflammasome is activated, a local inflammatory response occurs; inflammatory cells such as macrophages and T cells are recruited and activated; more ROS and cytokines are produced, leading to tissue injury (Zhang et al., 2012; Li et al., 2014). Therefore, ROS triggers NLRP3 inflammasome; an inflammatory response that occurs further increased the production of ROS; this may be a circular reaction. In addition to leukocytes, NLRP3 is also expressed in various non-professional immune cells and functions to regulate ROS production. Wu M. et al. (2018) demonstrated that NLRP3 knockdown blocks high glucose (HG)-induced TXNIP, NOX4 expression, and ROS generation in HK-2 cells. Wang et al. (2017) proved that inactivation of TXNIP–NLRP3 inflammasome pathway suppressed HG-induced ROS production, cell proliferation, and ECM accumulation in HBZY-1 cells. Song et al. (2018) indicated that knockdown of NLRP3 antagonized HG-induced epithelial–mesenchymal transition by inhibiting ROS production and phosphorylation of SMAD3, P38 MAPK, and ERK1/2. These studies have shown that NLRP3 could regulate the production of ROS, which was consistent with our findings. Thus, a dual function for NLRP3, both upstream and downstream of ROS, may exist in diverse cell types to modulate the cellular signaling processes. In the present study, we think ROS and NLRP3 also exist in this circular reaction relationship; overexpressing NLRP3 enhanced the inflammatory response and then increased the production of ROS under the ISO stimulation; knocking down NLRP3 reduced the inflammatory response and then decreased the production of ROS under the ISO stimulation. The NADPH oxidase family is the main source of ROS; increased NOX2 and NOX4 expression enhances NADPH oxidase activity and ROS production. Thus, NLRP3 caused changes in ROS derived from NADPH oxidase. Also, we demonstrated the regulatory relationship between ROS and HMGB1 with ROS scavenger NAC. However, we have not found a specific target for ROS to regulate HMGB1 nuclear translation and secretion. It needs much more experiments to explore the specific mechanism.

## Conclusion

In conclusion, our novel data extended previous observation that NLRP3 regulated the HMGB1 secretion via ROS production in cardiomyocytes and the secreted HMGB1 acting on CFs to promote their activation and proliferation leading to fibrosis, which supports the notion that NLRP3/ROS/HMGB1 axis may be a potential therapeutic target in the process of cardiac fibrosis.

## Data Availability Statement

The datasets generated for this study are available on request to the corresponding author.

## Ethics Statement

The animal study was reviewed and approved by the Animal Care and Use Committee of Renmin Hospital of Wuhan University.

## Author Contributions

CL and TH conceived the study design. QT and QW designed the experiments. CL, ZC, and YY analyzed the data. CL, ZC, QX, TH, SX, QY, and JZ performed the experiments. CL drafted the manuscript. QW and NL reviewed and revised the manuscript. All authors contributed to the article and approved the submitted version.

## Conflict of Interest

The authors declare that the research was conducted in the absence of any commercial or financial relationships that could be construed as a potential conflict of interest.

## Abbreviations

CFs: cardiac fibroblasts
DAMPs: danger-associated molecular patterns
DCFH − DA: dichlorofluorescein diacetate
ECM: extracellular matrix
HMGB1: high-mobility group box 1 protein
ISO: isoproterenol
KO: knockout
LVEDD: left ventricular end − diastolic diameter
LVESD: left ventricular end − systolic diameter
NC: negative control
NLRP3: nucleotide-binding oligomerization domain (NOD)-like receptor 3
NRVMs: neonatal rat ventricular myocytes
PAMPs: pathogen-associated molecular patterns
PYD: amino-terminal pyrin domain
PSR: picrosirius red staining
ROS: reactive oxygen species
RAGE: receptor for advanced glycation end products
TLR: toll-like receptors
WT: wild-type.
