# Supplementary material for: Nucleotide-Binding Oligomerization Domain-Like Receptor 3 Deficiency Attenuated Isoproterenol-Induced Cardiac Fibrosis via Reactive Oxygen Species/High Mobility Group Box 1 Protein Axis
Source: Front Cell Dev Biol. 2020 Aug 11;8:713. doi: 10.3389/fcell.2020.00713 (PMC7431462; doi:10.3389/fcell.2020.00713)
Supplement: Supplementary file 1 [file Image_1.pdf]

SUPPLEMENT

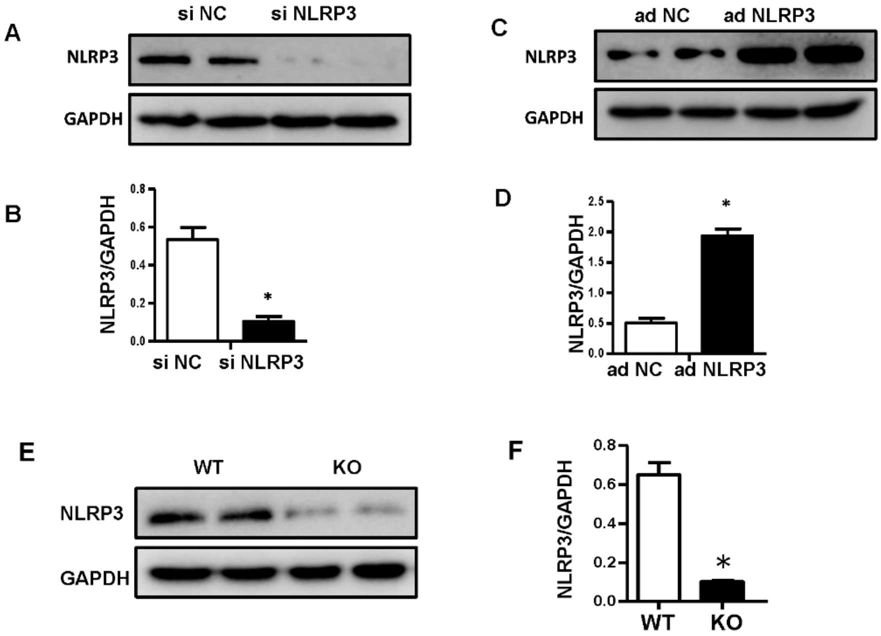

Figure S1. NLRP3 expression after transfecting with siNLRP3 or adNLRP3 in NRVMs and NLRP3 expression in WT and NLRP3-KO mouse hearts.

(A-B) The protein expression of NLRP3 in NRVMs transfected with siNLRP3. Western blot images (A) and quantitative results(B)(n=6). ＊ $P < 0.05$ versus si NC; (C-D) The protein expression of NLRP3 in NRVMs transfected with adNLRP3. Western blot images(C) and quantitative results(D) (n=6) ＊ $P < 0.05$ versus ad NC; (E-F) The protein expression level and quantitative results of NLRP3 in WT and NLRP3-KO mice. Western blot images(E) and quantitative results(F) (n=6). ＊ $P<0.05$ versus WT ;
